# Association between gestational age at threatened preterm birth diagnosis and incidence of preterm birth: the Japan Environment and Children’s Study

**DOI:** 10.1038/s41598-023-38524-9

**Published:** 2023-08-08

**Authors:** Tsuyoshi Murata, Hirotaka Isogami, Karin Imaizumi, Toma Fukuda, Hyo Kyozuka, Shun Yasuda, Akiko Yamaguchi, Akiko Sato, Yuka Ogata, Kosei Shinoki, Mitsuaki Hosoya, Seiji Yasumura, Koichi Hashimoto, Hidekazu Nishigori, Keiya Fujimori, the Japan Environment and Children’s Study (JECS) Group, Michihiro Kamijima, Shin Yamazaki, Yukihiro Ohya, Reiko Kishi, Nobuo Yaegashi, Koichi Hashimoto, Chisato Mori, Shuichi Ito, Zentaro Yamagata, Hidekuni Inadera, Takeo Nakayama, Tomotaka Sobue, Masayuki Shima, Hiroshige Nakamura, Narufumi Suganuma, Koichi Kusuhara, Takahiko Katoh

**Affiliations:** 1 Fukushima Regional Center for the Japan Environment and Children’s Study, 1 Hikarigaoka, Fukushima, 960-1295 Japan; 2 Department of Obstetrics and Gynecology, Fukushima Medical University School of Medicine, 1 Hikarigaoka, Fukushima, 960-1295 Japan; 3 Department of Pediatrics, Fukushima Medical University School of Medicine, 1 Hikarigaoka, Fukushima, 960-1295 Japan; 4 Department of Public Health, Fukushima Medical University School of Medicine, 1 Hikarigaoka, Fukushima, 960-1295 Japan; 5 Fukushima Medical Center for Children and Women, Fukushima Medical University, 1 Hikarigaoka, Fukushima, 960-1295 Japan; 6 Nagoya City University, Nagoya, Japan; 7 National Institute for Environmental Studies, Tsukuba, Japan; 8 National Centre for Child Health and Development, Tokyo, Japan; 9 Hokkaido University, Sapporo, Japan; 10 Tohoku University, Sendai, Japan; 11 Fukushima Medical University, Fukushima, Japan; 12 Chiba University, Chiba, Japan; 13 Yokohama City University, Yokohama, Japan; 14 University of Yamanashi, Chuo, Japan; 15 University of Toyama, Toyama, Japan; 16 Kyoto University, Kyoto, Japan; 17 Osaka University, Suita, Japan; 18 Hyogo Medical University, Nishinomiya, Japan; 19 Tottori University, Yonago, Japan; 20 Kochi University, Nankoku, Japan; 21 University of Occupational and Environmental Health, Kitakyushu, Japan; 22 Kumamoto University, Kumamoto, Japan

**Keywords:** Medical research, Epidemiology

## Abstract

We evaluated the association between gestational age at threatened preterm birth (TPTB) diagnosis and preterm birth (PTB) incidence using a nationwide birth cohort. Data of 94,236 women with singleton deliveries from the Japan Environment and Children’s Study (enrolled between 2011 and 2014) were analysed. Participants were divided based on parity and gestational age at TPTB diagnosis (22–24, 25–27, 28–30, 31–33, and 34–36 weeks). Multivariable logistic regression models were used to calculate the odds ratios (ORs) for PTB before 37 and 34 weeks in women from all groups, using participants without TPTB as the reference. The adjusted ORs for PTB before 37 weeks were the highest in the latest gestational age group in nulliparous and multiparous women without previous PTB, while those before 34 weeks were the highest in the earliest and latest gestational age group in multiparous women without previous PTB and in the earliest gestational age group in multiparous women with previous PTB. The association between gestational age at TPTB diagnosis and PTB incidence varies based on maternal parity and PTB before 37 or 34 weeks. Further studies with detailed clinical data and a unified TPTB diagnosis protocol are necessary to clarify this association.

## Introduction

Preterm births (PTBs) account for 75% of perinatal mortality cases and more than half of long-term morbidity cases^1–3^. The rate of PTB is increasing globally^4^. In Japan, the PTB rate had increased annually, from 4.1% in 1980 to 5.3% in 2000^5^, but since then it has remained stable. There has been no unified protocol for preventing PTB, and the research for strategies to reduce PTB is ongoing. Identifying women at risk remains a challenge^6^. Preventing PTB by addressing causal factors is paramount for improving neonatal outcomes. A detailed evaluation of women at risk of PTB is required for tailored treatment^2^. Thus, clarification of the association of maternal factors with PTB is required to reduce the incidence of PTB and improve the outcomes of infants.

Threatened preterm births (TPTBs) are a major risk factor for PTB, although 50% of women hospitalised for TPTB give birth at term^7^. TPTB is defined according to the clinical criteria of regular uterine contractions accompanied by a change in cervical dilatation, effacement, or both, or initial presentation with regular uterine contractions and cervical dilatation of at least 2 cm^7^. Multiple testing in women with TPTB has been proposed to detect women at risk of PTB, including measurement of cervical length (CL) and foetal fibronectin testing^6,8^. However, the clinical usefulness of these tests is primarily the ability to identify women who are least likely to deliver^6^. Moreover, TPTB has a wide range of characteristics, and the type of TPTB that presents the highest risks for PTB remains unknown^7^.

One characteristic of TPTB is gestational age at diagnosis, which is easy to identify. However, the clinical significance of this marker with stratified gestational ages has not been clarified. Moreover, the characteristics of mothers stratified by gestational ages at TPTB diagnosis remain unclear. We aimed to determine whether gestational age at TPTB diagnosis could predict the risk of PTB. A previous study reported that the risk of another PTB may be inversely correlated with the gestational age at the previous PTB^1^. Another previous study reported that early gestational age at TPTB diagnosis may be a risk factor for PTB^9^. Thus, we hypothesised that an earlier age at TPTB diagnosis is correlated with a higher risk of PTB based on these previous studies^1,9^.

We analysed the association between participants stratified by gestational age at diagnosis of TPTBs and the incidence of PTB before 37 and 34 weeks of gestation, using data from a nationwide Japanese birth cohort study. We also considered the difference of maternal parity in this analysis because nulliparous women without previous pregnancy details would need more information regarding the risk of PTB compared to multiparous women.

## Methods

### Study design

We analysed data from the Japan Environment and Children’s Study (JECS), which was a nationwide, government-funded, prospective birth cohort study started in January 2011 (participant enrolment: between January 2011 and March 2014) to investigate the effects of environmental factors on children’s health^10,11^. Briefly, the JECS was funded directly by the Ministry of the Environment, Japan and involved collaboration between the Programme Office (National Institute for Environmental Studies), Medical Support Centre (National Centre for Child Health and Development), and 15 Regional Centres (Hokkaido, Miyagi, Fukushima, Chiba, Kanagawa, Koshin, Toyama, Aichi, Kyoto, Osaka, Hyogo, Tottori, Kochi, Fukuoka, and South Kyushu/Okinawa)^10,11^. For inclusion in the JECS, expectant mothers had to meet the following criteria: (1) residence within the study area at the time of recruitment, with an expectation to continue residing in Japan in the foreseeable future; (2) expected due date between 1 August 2011 and mid-2014; and (3) the ability to participate in the study without difficulty (i.e. ability to comprehend the Japanese language and complete a self-administered questionnaire).

There were two modes of recruitment: (1) at the time of the first prenatal examination at co-operating health care providers; and (2) at local government offices that issued a pregnancy journal called the Maternal and Child Health Handbook to all expecting mothers in Japan before they received municipal services for pregnancy, delivery, and childcare. Pregnant women were contacted through co-operating health care providers and/or local government offices issuing Maternal and Child Health Handbooks, and those who were willing to participate were registered. Self-administered questionnaires, which were completed by the women during the first and second/third trimester, were used to collect information on demographic factors, medical history, physical and mental health, lifestyle, occupation, environmental exposures at home and in the workplace, housing conditions, and socioeconomic status^10,11^.

### Data collection

We used data released in October 2019 (dataset: jecs-ta-20190930). Participants with singleton pregnancies were included in the present study; however, women who had abortions, stillbirths, and missing information on exposures and outcomes were excluded from the analysis. Those with unreliable data regarding TPTB diagnosis and TPTB gestational age and missing data regarding maternal parity were also excluded. Parity was categorised into nulliparous and multiparous; multiparous women were further divided into two groups according to the presence of previous PTBs because women with previous PTBs had a 2.5-fold increased risk for their next pregnancy^1^. Moreover, we excluded cases with offspring chromosome abnormalities because those cases are related to PTBs.

### Exposure variables

TPTB was diagnosed by each co-operating health care provider and derived from medical record transcripts. There were no unified clinical criteria for diagnosis of TPTB in the JECS. However, in Japan, TPTB was typically diagnosed based on the presence of regular uterine contractions, or changes in CL or dilatation, which was different from other countries^7^. Gestational age was typically verified based on accurate ultrasound examinations conducted during the first trimester, while gestational age at diagnosis of TPTB was derived from medical record transcripts. Participants with TPTB were divided into five categories based on gestational age at diagnosis (22–24, 25–27, 28–30, 31–33, and 34–36 weeks of gestation).

### Main outcome measure

The main outcome measure was the incidence of PTB before 37 and 34 weeks of gestation. Gestational age at delivery was obtained from medical record transcripts. We also analysed PTB in participants without ischaemic placental disease (IPD) and chronic hypertension. IPD was based on the diagnosis of any of hypertensive disorders of pregnancy (HDP), small-for-gestational-age (SGA) infants, or placenta abruption (for which the data were derived from medical records). HDP was defined as persistently elevated blood pressure (≥ 140/90 mmHg) after 20 weeks of pregnancy in an otherwise normotensive woman^12^. SGA infants were defined as infants with birth weight < 1.5 standard deviations, corrected for parity, gestational age, and sex, based on the ‘New Japanese neonatal anthropometric charts for gestational age at birth’^13^. Although there is no consensus on the categorisation of PTBs^14^, cases of PTB with IPD would be the alternative for medically induced PTB.

### Confounding factors

The following were included as confounding factors: maternal age, body mass index before pregnancy, maternal smoking status, maternal alcohol consumption status, maternal educational status, annual household income, marital status, assisted reproductive technology, and high maternal Kessler 6 scores at the first half of pregnancy. There was no multicollinearity, which was considered to be present under the following conditions: an association between independent variables with correlation coefficient *r* > 0.8 and/or variance inflation factor > 10.

Maternal ages were categorised into < 20, 20–29, 30–39, and ≥ 40 years. Body mass index before pregnancy was categorised into < 18.5, 18.5–19.9, 20.0–22.9, 23.0–24.9, and ≥ 25.0 kg/m^2^. The participants were requested to provide information regarding their smoking status by selecting one of the following: ‘currently smoking’, ‘never’, ‘previously did, but quit before realising current pregnancy’, and ‘previously did, but quit after realising current pregnancy’. The participants who chose ‘currently smoking’ were included in the ‘smoking’ category, whereas others were included in the ‘non-smoking’ category. The participants were also requested to provide information regarding their alcohol consumption status by choosing one of the following: never drank, quit drinking before pregnancy, quit drinking during the early stage of pregnancy, and kept drinking during pregnancy^15^. Maternal participants who chose ‘kept drinking during pregnancy’ composed the drinking category; all other participants composed the non-drinking category. Maternal educational status was categorised into the following four groups according to the number of years of education completed: junior high school (< 10 years); high school (10–12 years); technical junior college, technical/vocational college, associate degree, or bachelor’s degree (13–16 years); and graduate degree (master’s/doctor’s) (≥ 17 years). The annual household income was categorised into four levels: < 2,000,000, 2,000,000–5,999,999, 6,000,000–9,999,999, and ≥ 10,000,000 JPY. High maternal Kessler 6 scores were defined as scores ≥ 13 points^16^. For each factor, ‘no answer’ was analysed as a single item.

### Statistical analysis

Women were stratified by the presence of TPTB, and maternal characteristics and obstetric outcomes were compared. Chi-square tests were performed to analyse the statistical differences of the ratio of PTBs between the groups, after the stratification of the participants based on parity. The ratio of intrauterine infections was also compared. Intrauterine infection was clinically diagnosed by physicians at each institution. There were no unified criteria for intrauterine infection in the JECS. Moreover, univariable and multivariable logistic regression models were used to calculate the crude odds ratios (cORs), adjusted ORs (aORs), and 95% confidence intervals (CIs) for the incidence of PTB before 37 and 34 weeks of gestation in participants in each category for gestational age at TPTB diagnosis (participants with diagnosis of TPTB at 34–36 gestational weeks were excluded from the analysis for PTB before 34 weeks), using participants without TPTB as the reference group. ORs were adjusted for potential confounding factors. We performed the same analyses excluding participants with IPD. Statistical analysis was performed using SPSS version 26 (IBM Corp., Armonk, NY, USA).

### Ethical approval

The JECS protocol was reviewed and approved by the Ministry of the Environment Institutional Review Board on Epidemiological Studies (approval no.: 100910001) and by the ethics committees of all participating institutions. The JECS was conducted in accordance with the Helsinki Declaration as well as with other national regulations and guidelines. Written informed consent was obtained from all participants.

## Results

**Figure 1:**
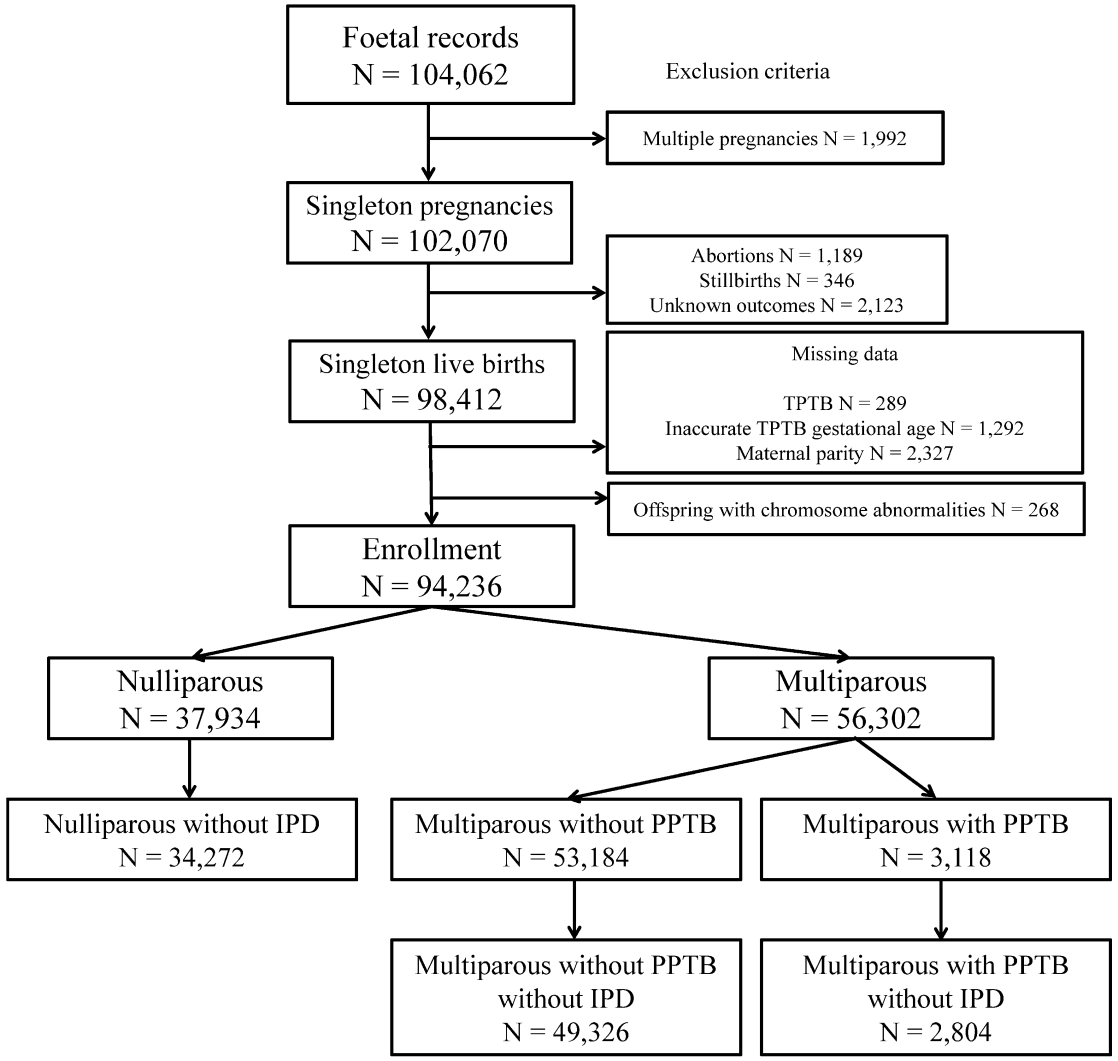
Enrolment flowchart. *TPTB* threatened preterm birth, *IPD* ischaemic placental disease, *PPTB* previous preterm birth.

The total number of foetal records in the JECS was 104,062. Overall, 94,236 participants met the inclusion criteria (Fig. 1).

Table 1 summarises the maternal characteristics and obstetric complications according to TPTB diagnosis status. The characteristics of participants with and without TPTB diagnosis did not differ significantly, except for the ratios of PTB before 37 and 34 weeks of gestation.

**Table 1:** Characteristics and outcomes of participants based on the presence of threatened preterm births.

	Total participants *N* = 94,236	
	TPTB	Non-TPTB
Variable	*N* = 17,525 (18.6%)	*N* = 76,711 (81.4%)
Maternal age, % (*n*)		
< 20 years	2.0 (352)	1.7 (1276)
20–29 years	43.4 (7608)	43.1 (33,071)
30–39 years	52.1 (9137)	52.7 (40,390)
≥ 40 years	2.4 (426)	2.6 (1970)
No answer	0.0 (2)	0.0 (4)
BMI before pregnancy, % (*n*)		
< 18.5 kg/m^2^	19.0 (3322)	15.4 (11,843)
18.5–19.9 kg/m^2^	25.8 (4514)	24.5 (18,814)
20.0–22.9 kg/m^2^	36.5 (6388)	37.9 (29,108)
23.0–24.9 kg/m^2^	9.5 (1665)	11.0 (8431)
≥ 25.0 kg/m^2^	9.3 (1625)	11.0 (8460)
No answer	0.1 (11)	0.1 (55)
Maternal smoking status, % (*n*)		
No	93.4 (16,365)	93.4 (71,613)
Yes	4.8 (844)	4.7 (3634)
No answer	1.8 (316)	1.9 (1464)
Maternal alcohol consumption status, % (*n*)		
No	94.8 (16,621)	94.8 (72,727)
Yes	2.6 (462)	2.8 (2155)
No answer	2.5 (442)	2.4 (1829)
Maternal educational status, % (*n*)		
< 10 years	5.0 (883)	4.7 (3612)
10–12 years	31.0 (5441)	30.9 (23,709)
13–16 years	60.5 (10,599)	60.8 (46,627)
≥ 17 years	1.2 (212)	1.5 (1123)
No answer	2.2 (390)	2.1 (1640)
Annual household income, % (*n*)		
< 2,000,000 JPY	5.2 (913)	5.2 (3960)
2,000,000–5,999,999 JPY	60.9 (10,668)	62.0 (47,568)
6,000,000–9,999,999 JPY	20.9 (3661)	20.4 (15,665)
≥ 10,000,000 JPY	3.9 (675)	3.9 (2990)
No answer	9.2 (1608)	8.5 (6528)
Marital status, % (*n*)		
Married	94.4 (16,551)	94.2 (72,242)
Not married	3.2 (564)	3.4 (2604)
Divorced	0.9 (150)	0.8 (634)
Husband died	0.0 (4)	0.0 (11)
No answer	1.5 (256)	1.6 (1220)
ART, % (*n*)		
No	96.7 (16,950)	96.8 (74,264)
Yes	3.0 (531)	2.8 (2154)
No answer	0.3 (44)	0.4 (293)
High maternal Kessler 6 scores, % (*n*)		
No	93.4 (16,369)	93.0 (71,345)
Yes	3.4 (598)	3.4 (2611)
No answer	3.2 (558)	3.6 (2755)
HDP, % (*n*)		
No	96.9 (16,980)	97.0 (74,381)
Yes	3.1 (545)	3.0 (2330)
SGA infants, % (*n*)		
No	94.9 (16,623)	94.9 (72,773)
Yes	5.0 (885)	5.0 (3827)
No answer	0.1 (17)	0.1 (111)
Placental abruption, % (*n*)		
No	99.8 (17,484)	99.8 (76,537)
Yes	0.2 (41)	0.2 (174)
HT, % (*n*)		
No	98.9 (17,336)	98.8 (75,761)
Yes	1.1 (189)	1.2 (950)
Preterm births before 37 weeks, % (*n*)		
No	89.0 (15,589)	97.0 (74,388)
Yes	11.0 (1936)	3.0 (2323)
Preterm births before 34 weeks, % (*n*)		
No	97.4 (17,067)	99.4 (76,275)
Yes	2.6 (458)	0.6 (436)

*BMI* body mass index, *JPY* Japanese yen, *ART* assisted reproductive technology, *SGA* small-for-gestational-age, *HT* hypertension, *HDP* hypertensive disorders of pregnancy.

Table 2 summarises the differences of outcomes based on the TPTB diagnosis status. The ratios of PTBs before 37 weeks in nulliparous and multiparous women without previous PTBs with TPTB diagnosis were maximised at 34–36 weeks of gestation. The PTB ratios before 37 weeks in multiparous participants with previous PTBs reached a plateau and did not show statistically significant results. There was no obvious trend in the increase of PTB ratios before 34 weeks according to the gestational age at TPTB diagnosis in nulliparous participants. The PTB ratios before 34 weeks in multiparous women without previous PTBs with TPTB diagnosis were maximised at 22–24 and 31–33 weeks of gestation. The ratios of PTBs before 34 weeks in multiparous women with previous PTBs with TPTB diagnosis were maximised at 22–24 weeks of gestation. The PTB ratios were much higher in multiparous women with previous PTBs compared with those in other groups. There were higher ratios of intrauterine infection in the early stage of TPTB in nulliparous women.

**Table 2:** The differences of outcomes based on the threatened preterm birth diagnosis status.

	Preterm births before 37 weeks, % (n)	Preterm births before 34 weeks, % (n)	Intrauterine infection, % (n)
Nulliparous women, *N* = 37,934 TPTB diagnosis, *N* = 6889			
22–24 weeks, *N* = 1748	8.9 (156)	2.6 (46)	2.1 (37)
25–27 weeks, *N* = 1238	9.0 (111)	3.1 (38)	1.1 (14)
28–30 weeks, *N* = 1643	8.8 (145)	2.8 (46)	1.4 (23)
31–33 weeks, *N* = 1479	12.2 (181)	2.8 (42)	0.8 (12)
34–36 weeks, *N* = 781	16.0 (125)		1.4 (11)
*p*-value	< 0.01	< 0.01	
Multiparous women without previous preterm births, *N* = 53,184 TPTB diagnosis, *N* = 9608			
22–24 weeks, *N* = 2203	7.9 (175)	2.7 (59)	0.6 (14)
25–27 weeks, *N* = 1626	8.0 (130)	2.5 (40)	0.9 (15)
28–30 weeks, *N* = 2245	7.5 (169)	2.1 (47)	0.5 (12)
31–33 weeks, *N* = 2151	10.0 (216)	2.9 (63)	0.4 (9)
34–36 weeks, *N* = 1383	16.5 (228)		1.0 (14)
*p*-value	< 0.01	< 0.01	
Multiparous women with previous preterm births, *N* = 3118 TPTB diagnosis, *N* = 1028			
22–24 weeks, *N* = 271	22.9 (62)	10.0 (27)	0.7 (2)
25–27 weeks, *N* = 197	31.0 (61)	7.6 (15)	0.5 (1)
28–30 weeks, *N* = 215	31.6 (68)	8.4 (18)	0.9 (2)
31–33 weeks, *N* = 214	32.7 (70)	7.9 (17)	0.9 (2)
34–36 weeks, *N* = 131	29.8 (39)		0.0 (0)
*p*-value	0.114	0.010	

Table 3 summarises the cORs, aORs, and 95% CIs for PTB before 37 weeks of gestation for nulliparous and multiparous women in each TPTB gestational age category. The aORs for PTBs in nulliparous and multiparous women with TPTB diagnosis increased in every category of gestational age at diagnosis. The aORs for PTBs in nulliparous and multiparous women without previous PTBs with TPTB diagnosis were maximised at 34–36 weeks of gestation. There was no obvious trend in the increase of aORs according to the gestational age at TPTB diagnosis in multiparous participants with previous PTBs.

**Table 3:** Crude and adjusted odds ratios for preterm births before 37 weeks were calculated in nulliparous and multiparous women with each gestational age at threatened preterm birth diagnosis status.

	Nulliparous		Multiparous without previous preterm births		Multiparous with previous preterm births	
	*N* = 37,934		*N* = 53,184		*N* = 3118	
	cOR (95% CI)	aOR (95% CI)	cOR (95% CI)	aOR (95% CI)	cOR (95% CI)	aOR (95% CI)
Reference group	Ref.	Ref.	Ref.	Ref.	Ref.	Ref.
22–24 weeks	3.12 (2.62–3.72)	3.13 (2.62–3.74)	3.21 (2.72–3.79)	3.22 (2.73–3.80)	2.31 (1.69–3.16)	2.42 (1.76–3.33)
25–27 weeks	3.14 (2.56–3.85)	3.19 (2.59–3.92)	3.24 (2.68–3.91)	3.20 (2.65–3.87)	3.49 (2.51–4.86)	3.77 (2.68–5.29)
28–30 weeks	3.08 (2.57–3.70)	3.16 (2.63–3.80)	3.03 (2.56–3.58)	3.07 (2.59–3.63)	3.60 (2.62–4.95)	3.86 (2.79–5.34)
31–33 weeks	4.44 (3.75–5.26)	4.63 (3.90–5.49)	4.16 (3.57–4.84)	4.21 (3.61–4.91)	3.78 (2.76–5.19)	4.00 (2.90–5.52)
34–36 weeks	6.07 (4.96–7.43)	6.37 (5.19–7.81)	7.35 (6.30–8.57)	7.47 (6.39–8.72)	3.30 (2.22–4.91)	3.46 (2.31–5.19)

Multivariable logistic regression analysis was adjusted for maternal age, body mass index before pregnancy, maternal smoking status, maternal alcohol consumption status, maternal educational status, annual household income, marital status, assisted reproductive technology, and high maternal Kessler 6 scores.

*CI* confidence interval, *cOR* crude odds ratio, *Ref.* reference, *aOR* adjusted odds ratio.

Table 4 summarises the cORs, aORs, and 95% CIs for PTB before 34 weeks of gestation for nulliparous and multiparous women in each TPTB gestational age category. The aORs for PTBs in nulliparous and multiparous women with TPTB diagnosis increased in every category of gestational age at diagnosis. The aORs for PTBs in nulliparous women reached a plateau. The aORs for PTBs in multiparous women without previous PTBs with TPTB diagnosis were maximised at 22–24 and 31–33 weeks of gestation. The aORs for PTBs in multiparous women with previous PTBs with TPTB diagnosis were maximised at 22–24 weeks of gestation.

**Table 4:** Crude and adjusted odds ratios for preterm births before 34 weeks were calculated in nulliparous and multiparous women with each gestational age at threatened preterm birth diagnosis status.

	Nulliparous		Multiparous without previous preterm births		Multiparous with previous preterm births	
	*N* = 37,934		*N* = 53,184		*N* = 3118	
	cOR (95% CI)	aOR (95% CI)	cOR (95% CI)	aOR (95% CI)	cOR (95% CI)	aOR (95% CI)
Reference group	Ref.	Ref.	Ref.	Ref.	Ref.	Ref.
22–24 weeks	4.07 (2.94–5.62)	4.01 (2.88–5.58)	6.86 (5.09–9.25)	6.94 (5.12–9.40)	3.95 (2.45–6.36)	4.36 (2.66–7.15)
25–27 weeks	4.76 (3.35–6.77)	4.86 (3.40–6.96)	6.29 (4.45–8.90)	6.16 (4.32–8.76)	2.94 (1.63–5.30)	3.29 (1.79–6.03)
28–30 weeks	4.33 (3.14–5.99)	4.60 (3.30–6.40)	5.33 (3.85–7.38)	5.59 (4.02–7.77)	3.26 (1.88–5.65)	3.33 (1.88–5.88)
31–33 weeks	4.40 (3.14–6.15)	4.81 (3.42–6.77)	7.53 (5.62–10.07)	8.02 (5.97–10.78)	3.08 (1.76–5.39)	3.24 (1.83–5.75)

Multivariable logistic regression analysis was adjusted for maternal age, body mass index before pregnancy, maternal smoking status, maternal alcohol consumption status, maternal educational status, annual household income, marital status, assisted reproductive technology, and high maternal Kessler 6 scores.

*CI* confidence interval, *cOR* crude odds ratio, *Ref.* reference, *aOR* adjusted odds ratio.

Table 5 summarises the cORs, aORs, and 95% CIs for PTB before 37 weeks of gestation in nulliparous and multiparous women with each TPTB gestational age category after the exclusion of IPD cases. The aORs for PTBs in nulliparous and multiparous women with TPTB diagnosis increased in every category of gestational age at diagnosis. The aORs for PTBs in nulliparous and multiparous women without previous PTBs with TPTB diagnosis were maximised at 34–36 weeks of gestation. The aORs for PTBs in multiparous participants with previous PTBs reached a plateau.

**Table 5:** Crude and adjusted odds ratios for preterm births before 37 weeks were calculated in nulliparous and multiparous women with each gestational age at threatened preterm birth diagnosis status, after exclusion of cases of ischaemic placental disease.

	Nulliparous		Multiparous without previous preterm births		Multiparous with previous preterm births	
	*N* = 34,272		*N* = 49,326		*N* = 2804	
	cOR (95% CI)	aOR (95% CI)	cOR (95% CI)	aOR (95% CI)	cOR (95% CI)	aOR (95% CI)
Reference group	Ref.	Ref.	Ref.	Ref.	Ref.	Ref.
22–24 weeks	3.50 (2.86–4.28)	3.48 (2.83–4.26)	3.73 (3.13–4.46)	3.70 (3.09–4.42)	3.07 (2.20–4.29)	3.09 (2.20–4.35)
25–27 weeks	4.00 (3.21–5.00)	4.02 (3.22–5.03)	3.85 (3.15–4.70)	3.82 (3.12–4.67)	4.30 (3.01–6.13)	4.51 (3.13–6.50)
28–30 weeks	3.75 (3.07–4.58)	3.78 (3.09–4.63)	3.41 (2.84–4.09)	3.42 (2.85–4.11)	4.70 (3.32–6.64)	4.84 (3.39–6.91)
31–33 weeks	5.65 (4.70–6.79)	5.80 (4.82–6.99)	4.70 (3.98–5.54)	4.74 (4.01–5.60)	4.72 (3.37–6.62)	4.91 (3.47–6.93)
34–36 weeks	8.05 (6.49–9.98)	8.19 (6.60–10.18)	8.92 (7.59–10.50)	9.00 (7.64–10.61)	4.47 (2.94–6.79)	4.60 (3.01–7.04)

Multivariable logistic regression analysis was adjusted for maternal age, body mass index before pregnancy, maternal smoking status, maternal alcohol consumption status, maternal educational status, annual household income, marital status, assisted reproductive technology, and high maternal Kessler 6 scores.

*CI* confidence interval, *cOR* crude odds ratio, *Ref.* reference, *aOR* adjusted odds ratio.

Table 6 summarises the cORs, aORs, and 95% CIs for PTB before 34 weeks of gestation for nulliparous and multiparous women in each TPTB gestational age category after the exclusion of IPD cases. The aORs for PTBs in nulliparous and multiparous women with TPTB diagnosis increased in every category of gestational age at diagnosis. There was no obvious trend in the increase of aORs according to the gestational age at diagnosis of TPTB in nulliparous participants. The aORs for PTBs in multiparous women without previous PTBs with TPTB diagnosis were maximised at 22–24 and 31–33 weeks of gestation. The aORs for PTBs in multiparous women with previous PTBs with TPTB diagnosis were maximised at 22–24 weeks of gestation.

**Table 6:** Crude and adjusted odds ratios for preterm births before 34 weeks were calculated in nulliparous and multiparous women with each gestational age at threatened preterm birth diagnosis status, after exclusion of cases of ischaemic placental disease.

	Nulliparous		Multiparous without previous preterm births		Multiparous with previous preterm births	
	*N* = 34,272		*N* = 49,326		*N* = 2804	
	cOR (95% CI)	aOR (95% CI)	cOR (95% CI)	aOR (95% CI)	cOR (95% CI)	aOR (95% CI)
Reference group	Ref.	Ref.	Ref.	Ref.	Ref.	Ref.
22–24 weeks	7.10 (4.82–10.47)	6.91 (4.65–10.26)	10.46 (7.44–14.71)	10.31 (7.28–14.61)	6.57 (3.84–11.22)	7.49 (4.28–13.13)
25–27 weeks	9.39 (6.31–13.97)	9.41 (6.27–14.12)	9.42 (6.36–13.96)	9.48 (6.35–14.14)	5.10 (2.71–9.60)	5.75 (2.98–11.13)
28–30 weeks	6.97 (4.69–10.36)	7.22 (4.82–10.80)	7.76 (5.34–11.27)	8.07 (5.52–11.80)	5.31 (2.86–9.87)	5.09 (2.66–9.74)
31–33 weeks	7.67 (5.14–11.46)	8.23 (5.48–12.36)	12.28 (8.85–17.04)	13.02 (9.33–18.17)	4.63 (2.46–8.71)	4.89 (2.56–9.36)

Multivariable logistic regression analysis was adjusted for maternal age, body mass index before pregnancy, maternal smoking status, maternal alcohol consumption status, maternal educational status, annual household income, marital status, assisted reproductive technology, and high maternal Kessler 6 scores.

*CI* confidence interval, *cOR* crude odds ratio, *Ref.* reference, *aOR* adjusted odds ratio.

## Discussion

The present study revealed that the association between gestational age at TPTB diagnosis and the incidence of PTB varies based on maternal parity with a history of previous PTB and PTB before 37 or 34 weeks. Latest gestational age at diagnosis of TPTBs was associated with a higher incidence of PTBs before 37 weeks in nulliparous and multiparous women without previous PTBs. Meanwhile, earliest gestational age at TPTB diagnosis was associated with a higher incidence of PTBs before 34 weeks in multiparous women. The same tendency was confirmed in nulliparous and multiparous women after excluding the cases of patients with IPD. To the best of our knowledge, this is the first study to show the association between gestational age at TPTB diagnosis and the incidence of PTBs in a nationwide birth cohort with stratification of maternal parity and history of previous PTBs.

We speculate that one of the reasons for the difference in the results between PTB before 37 and 34 weeks of gestation could stem from differences in PTB aetiology between the early and late stages. The major aetiologies of PTB are infection in the early stage and hormonal changes in the late stage^17^. In this study, we identified higher ratios of intrauterine infection in the early stage of TPTB in nulliparous women; however, the number of participants with intrauterine infection was too small to draw a definitive conclusion that differences in PTB aetiology between the early and late stages would cause differences in the association between gestational age at diagnosis of TPTB and the incidence of PTBs. Regarding PTBs before 37 weeks, later TPTB diagnosis might be a predictor for PTB incidence. There would be a shorter CL and wider cervical dilatation at the time of TPTB diagnosis in the later TPTB group than in the early TPTB group because shorter CL and wider cervical dilatations are physiologically induced as gestational age progresses. Therefore, TPTB diagnosis at 34–36 weeks of gestation might be generally diagnosed based on uterine contractions and might lead to a higher rate of onset of labour immediately after TPTB diagnosis because cervical dilatation is strongly associated with the latency of pregnancy after TPTB diagnosis^9,18^. However, it should be noted that the majority of PTBs occur at a later gestational age; in particular, 73.2% of PTB cases occur at 34–36 weeks^19^. Regarding PTBs before 34 weeks, earliest TPTB diagnosis might be a predictor for PTB incidence in multiparous women. This is consistent with the findings of a previous study^9^ and our hypothesis. This information would be useful for multiparous women, because both clinicians and women may be predisposed to recommend or seek evaluation at a potentially lower threshold of symptoms when a previous PTB has occurred in multiparous women^20^. However, in clinical settings, the requirement for predicting of PTBs is greater in nulliparous than in multiparous women, because multiparous women already have important information concerning the prediction of PTBs (i.e. previous PTBs). The clear explanation of the plateau aORs for PTBs in nulliparous women and maximised aORs for PTBs in multiparous women without previous PTBs with TPTB diagnosis at 31–33 weeks of gestation is lacking. Therefore, the utility of gestational age at TPTB diagnosis for the prediction of PTBs before 37 and 34 weeks is questionable and needs further validation.

Moreover, the difference between PTB before 37 and 34 weeks of gestation would be caused by the differences in methods used for diagnosing TPTB in Japan. There may be some differences in the criteria for patients with TPTB in Japan compared to those in other countries^7^; reports from other countries have shown that hospital admissions for threatened preterm labour was approximately 9% (129 women for TPTB diagnosis at 24–32 weeks and 105 women for TPTB diagnosis above 33 weeks among 2,534 participants)^20^. More participants were diagnosed with TPTB in the JECS compared to those in this previous study^20^. In Japan, many pregnant women would have been diagnosed as having TPTB based on just slight uterine contractions or short CL, and those patients with TPTB under these criteria are often treated with long-term maternal ritodrine hydrochloride administration^21^. The inconsistency of TPTB diagnosis in Japan might have led to the discrepancy between the present results and our hypothesis. Moreover, the higher prevalence of TPTB in the JECS would be partially attributed to the fact that we did not consider the difference between outpatients and hospitalisations. Further studies with unified protocols for diagnosing TPTB are warranted to clarify the true association between gestational age at TPTB diagnosis and PTB incidence.

The present study has some limitations. First, several clinical factors may affect the results. We did not account for detailed clinical scenarios, such as CL, cervical dilatation, uterine contractions, foetal fibronectin testing, genital bleeding during pregnancy, amniotic fluid levels, laboratory data including inflammatory cytokines, and treatments including tocolysis and cerclage. Further studies are required to clarify the association between gestational age at TPTB diagnosis and the incidence of PTB based on these clinical factors. Second, there is a possibility of selection bias, as several participants who had missing data were excluded. Although no significant differences were noted in the characteristics between those included and excluded from the analysis owing to missing data (data not shown), careful interpretation is needed based on considerations of these potential biases.

## Conclusions

The association between gestational age at diagnosis of TPTB and the incidence of PTB varies based on maternal parity with a history of previous PTB and PTB before 37 or 34 weeks. This variety would be caused by differences in PTB aetiology between the early and late stages of pregnancy, differences in patient characteristics between the early and late stages of TPTB diagnosis, and differences in methods used for diagnosing TPTB in Japan compared to those in other countries. The utility of gestational age at TPTB diagnosis to predict PTB incidence in a clinical setting remains unclear. Further studies with detailed clinical data and a unified TPTB diagnosis protocol are necessary to clarify the association between gestational age at TPTB diagnosis and PTB incidence.

## Data Availability

Data are unsuitable for public deposition due to ethical restrictions and legal framework of Japan. It is prohibited by the Act on the Protection of Personal Information (Act No. 57 of 30 May 2003, amendment on 9 September 2015) to publicly deposit the data containing personal information. Ethical Guidelines for Epidemiological Research enforced by the Japan Ministry of Education, Culture, Sports, Science and Technology and the Ministry of Health, Labour and Welfare also restrict the open sharing of the epidemiologic data. All inquiries about access to data should be sent to: jecs-en@nies.go.jp. The person responsible for handling enquiries sent to this e-mail address is Dr. Shoji F. Nakayama, JECS Programme Office, National Institute for Environmental Studies.

## Abbreviations

aOR: Adjusted odds ratio
CI: Confidence interval
CL: Cervical length
cOR: Crude odds ratio
HDP: Hypertensive disorders of pregnancy
IPD: Ischaemic placental disease
JECS: Japan Environment and Children’s Study
PTB: Preterm birth
SGA: Small-for-gestational-age
TPTB: Threatened preterm birth
